# Differing Behaviors Around Adult Nonmedical Use of Prescription Stimulants and Opioids: Latent Class Analysis

**DOI:** 10.2196/46742

**Published:** 2023-09-20

**Authors:** Karilynn M Rockhill, Richard Olson, Richard C Dart, Janetta L Iwanicki, Joshua C Black

**Affiliations:** 1 Rocky Mountain Poison and Drug Safety Denver Health and Hospital Authority Denver, CO United States

**Keywords:** stimulant misuse, opioid misuse, high-dimensional analysis, latent class analysis, general population, drug overdose, opioid, drug use pattern, nonmedical use of prescription stimulant, substance abuse, drugs, adulthood

## Abstract

**Background:**

The availability of central nervous system stimulants has risen in recent years, along with increased dispensing of stimulants for treatment of, for example, parent-reported attention-deficit/hyperactivity disorder in children and new diagnoses during adulthood. Typologies of drug use, as has been done with opioids, fail to include a sufficient range of behavioral factors to contextualize person-centric circumstances surrounding drug use. Understanding these patterns across drug classes would bring public health and regulatory practices toward precision public health.

**Objective:**

The objective of this study was to quantitatively delineate the unique behavioral profiles of adults who currently nonmedically use stimulants and opioids using a latent class analysis and to contrast the differences in findings by class. We further evaluated whether the subgroups identified were associated with an increased Drug Abuse Screening Test-10 (DAST-10) score, which is an indicator of average problematic drug use.

**Methods:**

This study used a national cross-sectional web-based survey, using 3 survey launches from 2019 to 2020 (before the COVID-19 pandemic). Data from adults who reported nonmedical use of prescription stimulants (n=2083) or prescription opioids (n=6127) in the last 12 months were analyzed. A weighted latent class analysis was used to identify the patterns of use. Drug types, motivations, and behaviors were factors in the model, which characterized unique classes of behavior.

**Results:**

Five stimulant nonmedical use classes were identified: amphetamine self-medication, network-sourced stimulant for alertness, nonamphetamine performance use, recreational use, and nondiscriminatory behaviors. The drug used nonmedically, acquisition through a friend or family member, and use to get high were strong differentiators among the stimulant classes. The latter 4 classes had significantly higher DAST-10 scores than amphetamine self-medication (*P*<.001). In addition, 4 opioid nonmedical use classes were identified: moderate pain with low mental health burden, high pain with higher mental health burden, risky behaviors with diverse motivations, and nondiscriminatory behaviors. There was a progressive and significant increase in DAST-10 scores across classes (*P*<.001). The potency of the opioid, pain history, the routes of administration, and psychoactive effect behaviors were strong differentiators among the opioid classes.

**Conclusions:**

A more precise understanding of how behaviors tend to co-occur would improve efficacy and efficiency in developing interventions and supporting the overall health of those who use drugs, and it would improve communication with, and connection to, those at risk for severe drug outcomes.

## Introduction

### Background

Central nervous system stimulants are treatments for a variety of illnesses. There are pharmaceutical treatments for attention-deficit/hyperactivity disorder (ADHD), such as treatments using amphetamine and methylphenidate [1], and for narcolepsy, shift work sleep disorder, and obstructive sleep apnea, such as treatment using modafinil [2]. In recent years, the availability of these drugs has risen. Among children and adolescents aged 3 to 17 years, the prevalence of parent-reported ADHD had risen steadily to 9.6% in 2018 [3], whereas dispensing rates for stimulants remained stable from 2014 through 2019 [4]. Among adults, the prevalence of diagnosed ADHD was estimated at 2.5% in 2009 [1], and the rates of dispensing, particularly among female individuals, were on the rise from 2014 to 2019 [4]. Heterogeneity in the clinical presentation of ADHD among adults makes clinical diagnoses more difficult during adulthood than during adolescence [5]. In the United States, an estimated 536,000 (0.2%) individuals aged ≥12 years used modafinil in 2020 [6]. Given the effects of prescription stimulants on the central nervous system, all types have the potential to be misused or abused [7], and the diversion of prescription stimulants is well documented [8].

In 2020, in the United States, an estimated 5.1 million (1.8%) people aged ≥12 years misused a prescription stimulant, 5.2 million used cocaine, and 2.5 million used methamphetamine. However, 50% more people initiated prescription stimulant misuse than cocaine, and 400% more people misused prescription stimulants than methamphetamine [6]. Prescription stimulant misuse, or nonmedical use, has been documented in several sectors of society, particularly among younger adults and adolescents [8,9]. Most of those who misuse prescription stimulants are not novel drug users; the majority also report using other recreational drugs or misuse of other prescription drugs (95.3%) and report that their misuse behaviors were preceded by other drug use (77.6%) [10]. In addition, mortality involving illicit and prescription psychostimulants has risen dramatically, with 22.9% of drug overdose deaths involving a stimulant in 2019 [11], and amphetamine-involved deaths are also on the rise, albeit at lower levels [12]. As drug use behaviors shift, and new medications become more prevalent, our characterization of behavioral patterns should also change.

Typologies of drug use among persons who use opioids have been studied using a latent class analysis (LCA), with a substantial emphasis on understanding polysubstance use [13,14]. However, few studies have investigated the behavioral constructs that might underlie opioid use. Apart from accounting for injection or inhalation, typology analyses involving opioids have not factored in behavioral indicators such as the source of the drug or reasons for use, although the association of behavioral indicators with the severity of drug use is well known [15-17]. Precision public health [18], which heralds a move to providing the right intervention to the right individual, is needed and requires an understanding of the diversity in drug use patterns in real-world settings. Drug class–specific use patterns may present as heterogeneous typologies; in addition, the typologies of use are not expected to be similar among drug classes. The behavioral typologies of both stimulants and opioids are understudied, and distinguishing behavioral patterns among drug classes would be a step toward person-centric precision public health.

### Objectives

This study aimed to delineate more nuanced behavioral patterns among those who use prescription stimulants and opioids using a national survey in the United States and an LCA. An objective of this study was to compare the differences in how the latent classes manifested in each of the stimulant and opioid classes across a similar set of behavioral indicators. This approach allows the data-driven discovery of use patterns across many novel behavioral indicators, which broadens the understanding of the typologies of use for both drug classes. A direct comparison between prescription stimulant and opioid typologies from the same data source and study period contrasts important similarities and differences between these psychoactive substances [13].

## Methods

### Study Population

This study used cross-sectional data from the Researched Abuse, Diversion and Addiction-Related Surveillance System’s Survey of the Nonmedical Use of Prescription Drugs (NMURx) program. Detailed methods for the NMURx program are provided elsewhere [19]. Briefly, this program conducts 2 launches of the survey per year to a web-based panel targeting the general adult population (aged ≥18 years); respondents can only participate once per year. The survey includes information about the individual’s characteristics and risk factors, as well as their motivations and behaviors surrounding prescription drug use. The questions focus on major psychoactive drug classes, including stimulants and opioids. Respondents are surveyed based on a nonprobability-based quota sampling collection mechanism. Exclusion criteria are then applied to remove respondents who demonstrate careless or inattentive responses. Finally, the sample is weighted using a generalized calibration weighting scheme. This approach has been shown to generate reliable and valid drug use estimates in the United States among adults [19,20]. This study used both survey launches from 2019 and the first launch from 2020; data collection was completed before widespread disruption to daily life in March 2020 from the COVID-19 pandemic.

### Measures

This study focused on those who have nonmedically used prescription stimulants or prescription opioids in the last 12 months. Nonmedical use was defined as a prescription drug “use in a way not directed by a health care professional.” The prescription stimulants of interest were amphetamine, methylphenidate, and modafinil. Given the many different types of prescription opioids, these were classified by morphine milligram equivalent conversion factors into 4 indicator variables: low-potency opioids (codeine, dihydrocodeine, tramadol, and tapentadol), medium-potency opioids (hydrocodone, morphine, and oxycodone), high-potency opioids (fentanyl, hydromorphone, and oxymorphone), and opioids used commonly in opioid use disorder maintenance therapy (buprenorphine and methadone) [21]. Drug use behaviors were classified as indicator variables. Reasons for nonmedical use are tailored to each drug class; a full list of reasons by class is provided in Multimedia Appendix 1. The routes of administration included swallowing, other oral methods (including crushing, chewing, or dissolving in mouth), inhaling (snorting or smoking), and injecting the medication. The sources of the prescription included a valid prescription for oneself, friend, or family member; dealers; or another form of diversion (a forged prescription; stolen from pharmacy, clinic, or hospital; or purchased without a prescription abroad or on the internet). Finally, nonpharmaceutical stimulant (cocaine, crack cocaine, methamphetamine, illicit amphetamine, or 3,4-methylenedioxy-methamphetamine [MDMA]) and nonpharmaceutical opioid (heroin or nonpharmaceutical fentanyl) use in the last year were defined as nonpharmaceutical drug use indicator variables for each of the stimulant and opioid models. All drug use indicators were included as predictors in the LCA models.

Other demographic and risk factors were included to describe the sample and the resultant latent classes. In particular, the Drug Abuse Screening Test-10 (DAST-10) was used to provide a quantitative score for class-agnostic severity of drug use [22]. This self-administered tool is a 10-item questionnaire that is not substance specific; hence, scores can be compared across populations. A score of ≥3 on the DAST-10 discriminates well for people with lifetime drug use disorders with or without diagnoses [23]. Participation in a drug treatment program in the last 12 months for drug use disorders involving prescription or illegal substances was self-reported.

### Ethical Considerations

The NMURx program study protocol has received ongoing approval from the Colorado Multiple Institutional Review Board since 2016 (#16-0922). Participants consented to be surveyed (Multimedia Appendix 2) and were compensated in points, which could be redeemed for gift cards at commercial vendors valued up to US $3. Privacy in the survey is protected by a certificate of confidentiality from the National Institutes of Health, and personally identifiable information was not collected by the researchers.

### Statistical Analysis

Demographics and national prevalence of drug use, as well as nonmedical use for each prescription drug and 95% CI, were estimated among all adults in the United States using calibration weights. Among those who nonmedically used a prescription stimulant or opioid, the percentage and 95% CI for each drug, reason, route, and source were estimated separately.

The populations of those who nonmedically used prescription stimulants or opioids were independently explored using an LCA. Other studies that have explored the patterns of drug use behaviors have taken similar approaches [13,14]. This multivariable technique assumes that the study population consists of several subgroups engaging in a mix of distinct behaviors that are defined by observed characteristics [24]. These subgroups were latent, meaning that they were not directly observed and were instead discerned from individual behaviors. Subgroups derived by the data can differ between the 2 prescription drug types; however, naming the identified subgroups was qualitative and dictated by the researchers based on the patterns observed.

To develop the LCA models, several exploratory subsets of the model indicators were used from the categories of drug type, reason for nonmedical use, route of administration, and source. The selection process was performed to improve model performance and interpretability [24,25]. There was no a priori number of latent classes specified; therefore, models with different latent classes were tested and compared with the Bayesian information criterion (BIC) and other fit statistics. Degrees of freedom for the G^2^ likelihood ratio test were calculated from the fitted model, which is the number of cells in a contingency table representing permutations of behaviors minus the number of parameters estimated minus 1. Other diagnostic criteria evaluated were the smallest class count (sample size of >50 or >5% of the sample), entropy (>0.8), and average latent class posterior membership probability (>0.9) [25]. LCA models that did not converge are not shown. The NMURx program calibration weights were incorporated into the models using a pseudolikelihood estimation approach.

The latent class modeling results presented are the gamma (γ) parameters (the latent class membership probabilities or prevalence estimates) and rho (ρ) parameters (the item-response probabilities given latent class membership). Individuals were assigned to a latent class with the highest probability based on the Bayes theorem, given the observed data [24]. The distributions of demographic and health factors were stratified by latent class. Differences in these characteristics across class membership were assessed using chi-square tests for categorical variables and ANOVA for continuous variables; *P* values were adjusted using the Holm correction for multiple comparisons.

Finally, it was hypothesized that subgroups with more advanced drug use behaviors would be associated with an increased DAST-10 score, as an indicator of average problematic drug use, and with attending drug use treatment in the last 12 months. When evaluating distal outcomes, we used the *naive approach*, where the latent class assignment was used in subsequent models without including the error or bias associated with class membership probabilities [26,27]. Other methods for accounting for this error or bias have been published, but the naive approach allowed the incorporation of the NMURx program weighting scheme. This resulted in an acceptable trade-off between potentially attenuated effect sizes and the representative modeling results of the general population. Both crude and adjusted models were assessed. The association of class membership with mean DAST-10 score was modeled with a linear regression controlling for sex, age, race (Black, White, or other), ethnicity, and tobacco use in the last 12 months to adjust for confounding. The odds of drug use treatment in the last 12 months were modeled with logistic regression controlling for the same variables plus a history of acute or chronic pain.

All analyses were conducted in SAS (version 9.4; SAS Institute) using the *PROC LCA* package [28].

## Results

### Study Sample

Across the study period, there were 89,383 surveys representing 87,786 unique individuals between the 2 survey years. Of the 87,786 unique individuals, 2083 (2.37%) indicated nonmedical use of stimulants, and 6127 (6.98%) indicated nonmedical use of opioids in the last 12 months. In the study, 1.79% (1597/89,383) surveys were from repeat individuals; this was considered ignorable in the analysis.

The national prevalence among adults for any prescription stimulant use in the last 12 months was 5% (95% CI 4.9%-5.2%), including the use of amphetamine (4%, 95% CI 3.8%-4.1%), methylphenidate (1%, 95% CI 1.0%-1.1%), and modafinil (0.7%, 95% CI 0.6%-0.7%). The national prevalence of nonmedical use of prescription stimulants was 1.9% (95% CI 1.8%-2.0%), including amphetamine (1.6%, 95% CI 1.5%-1.6%), methylphenidate (0.3%, 95% CI 0.3%-0.4%), and modafinil (0.2%, 95% CI 0.2%-0.3%). The national prevalence of nonmedical use of any prescription opioid was 5.5% (95% CI 5.4%-5.7%), including medium-potency opioids (3.4%, 95% CI 3.2%-3.5%), low-potency opioids (2.9%, 95% CI 2.8%-3.1%), high-potency opioids (0.7%, 95% CI 0.6%-0.8%), and maintenance opioids (0.7%, 95% CI 0.6%-0.7%).

Overall, compared with the general population, the adults who nonmedically used prescription drugs were younger and consisted of higher proportions of male individuals, individuals who identified as Hispanic, those who had never married, and current students or health care professionals (Table S1 in Multimedia Appendix 1). In the last 12 months, there was also a higher proportion of alcohol use, an overnight stay in the hospital, and receipt of counseling for alcohol or drug use. The proportions for the lifetime history of 10 mental health diagnoses were ≥2-fold higher among those who had nonmedically used prescription medications. Finally, the average DAST-10 score was higher among those who reported nonmedical use of prescription stimulants compared with those who reported nonmedical use of opioids or the total population.

### Subgroups of Stimulant Nonmedical Use

#### Item Response and Model Fit Overview

Among adults who nonmedically used prescription stimulants, amphetamine was the most common drug (79.9%), followed by nonpharmaceutical stimulants (35.3%; Table S2 in Multimedia Appendix 1). The most common reasons for nonmedical use included to focus or get work done (62.8%) and to stay awake or be alert (59.6%), whereas in the case of a little more than one-third of adults, the reasons included for enjoyment and to get high (35.2%). A friend or family member was the most common source (55.4%) of the prescription stimulant, followed by individuals who used their own valid prescriptions (41.5%).

**Figure 1:**
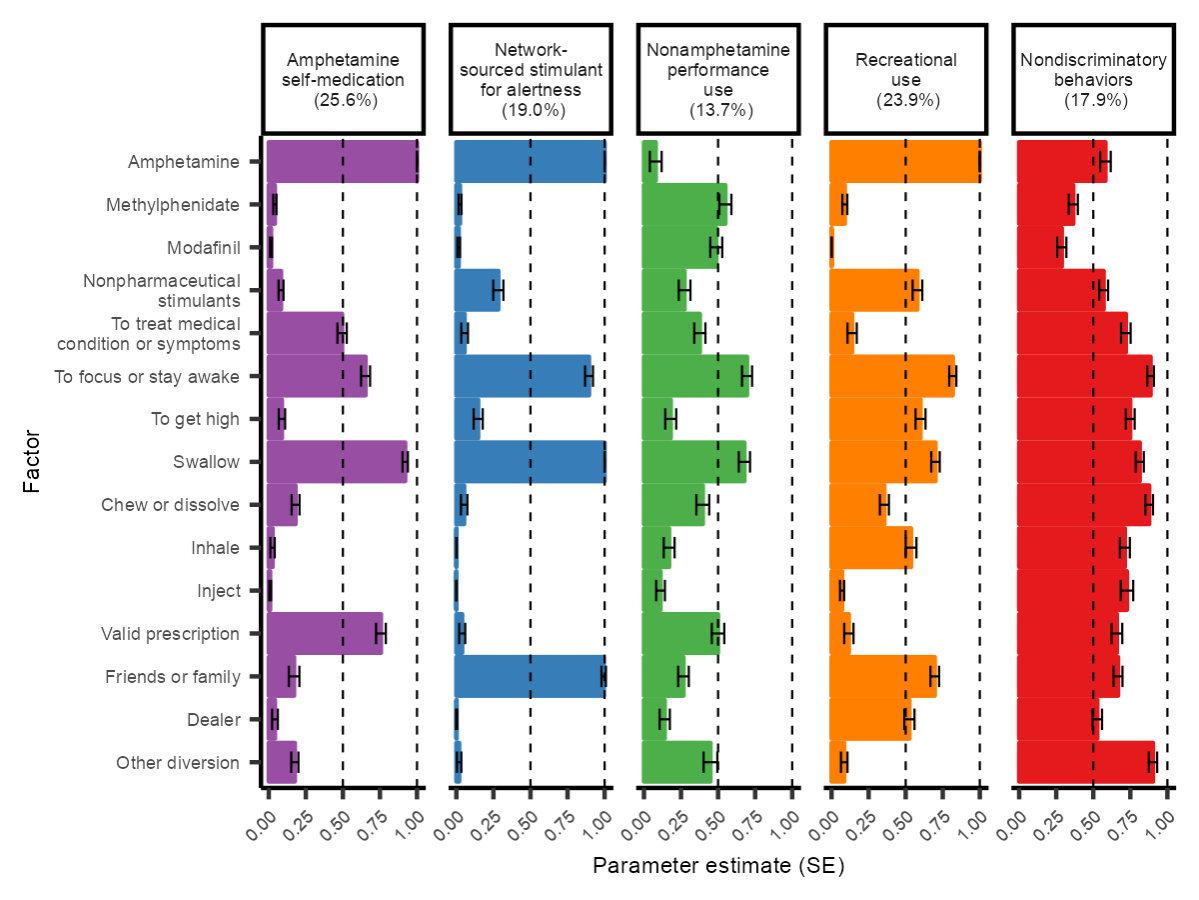
Stimulant nonmedical use latent class item-response probabilities.

The stimulant LCA resulted in up to 5 latent classes across converged models, and the 5-class model had the lowest BIC and appropriate fit statistics (Tables 1 and 2). Full item-response probabilities and 95% CI for each observed variable across the classes are included in Table S3 in Multimedia Appendix 1. Among adults nonmedically using prescription stimulants, the interpretation of the 5 latent classes and their prevalence estimates (Figure 1; Table S4 in Multimedia Appendix 1) are provided in the following subsections.

**Table 1:** Latent class analysis model evaluation (model fit criteria) by drug group.

Drug group and number of classes			Model fit criteria						
			BIC^a^		AIC^b^		G^2^^c^(*df*)		Log-likelihood
**Nonmedical use of prescription stimulants**									
	1	11,168.9		11,084.3		11,054.3 (32,752)		–17,687.9	
	2	8682.6		8507.7		8445.7 (32,736)		−16,383.6	
	3	7439.6		7174.5		7080.5 (32,720)		−15,701.0	
	4	6990.6		6635.2		6509.2 (32,704)		−15,415.4	
	5	6518.2		6072.5		5914.5 (32,688)		−15,118.0	
**Nonmedical use of prescription opioids**									
	1	34,598.9		34,484.7		34,450.7 (131,054)		−56,757.0	
	2	24,098.1		23,862.8		23,792.8 (131,036)		−51,428.1	
	3	22,355.3		21,999.1		21,893.1 (131,018)		−50,478.2	
	4	20,671.7		20,194.6		20,052.6 (131,000)		−49,557.9	

^a^BIC: Bayesian information criterion.

^b^AIC: Akaike information criterion.

^c^Likelihood ratio chi-square statistic for null hypothesis.

**Table 2:** Latent class analysis model evaluation (diagnostic criteria) by drug group.

Drug group and number of classes			Diagnostic criteria						
			Smallest class count, n (%)		Smallest class weighted prevalence (%)		Entropy		Average latent class posterior probability
**Nonmedical use of prescription stimulants (n=2083)**									
	1	2083 (100)		100		1.00		1.00	
	2	844 (40.5)		32.4		0.78		0.94	
	3	627 (30.1)		32		0.81		0.92	
	4	362 (17.4)		21.7		0.83		0.91	
	5	320 (15.4)		13.4		0.85		0.91	
**Nonmedical use of prescription opioids (n=6127)**									
	1	6127 (100)		100		1.00		1.00	
	2	1999 (32.6)		28.2		0.87		0.96	
	3	1336 (21.8)		24.5		0.88		0.95	
	4	1215 (19.8)		19.2		0.85		0.92	

#### Amphetamine Self-Medication

This class (25.6%) was defined by amphetamine users (ρ=1.00) with an extremely low probability of other stimulant use. This class had a moderate probability of nonmedically using amphetamine for addressing medical conditions or symptoms (ρ=0.49) or alertness (ρ=0.65) as well as high probabilities of swallowing the medication (ρ=0.92) and sourcing it through a valid prescription (ρ=0.76). This class had almost 2-fold higher proportions of ADHD diagnoses than other classes and similarly high proportions of anxiety disorders. Nonpharmaceutical drug use was lowest among the classes, and the prevalence was largely comparable with the general population prevalence estimates. This class had low but nonzero probability of nonmedical use of low- and medium-potency opioids.

#### Network-Sourced for Alertness

This class (19%) was also defined by amphetamine use (ρ=1.00), with moderate nonpharmaceutical stimulant use (ρ=0.28). This class had high probabilities of nonmedically using stimulants to stay awake or be alert (ρ=0.89) and swallowing the medication (ρ=1.00). This class almost exclusively sourced the stimulants through friends and family networks (ρ=0.99), with very low probability of using a valid prescription or sourcing from dealers. This class had the lowest proportion of diagnosed ADHD despite high amphetamine use. Cocaine and MDMA use were also elevated in this group. As in the amphetamine self-medication group, this class had low but nonzero probability of nonmedical use of low- and medium-potency opioids.

#### Nonamphetamine Performance Use

This class (13.7%) was defined by higher probabilities of prescription methylphenidate (ρ=0.55) and modafinil (ρ=0.49) use and distinctly not amphetamine use (ρ=0.08). This class had high probabilities of nonmedically using the stimulants for alertness (ρ=0.70), swallowing the medication (ρ=0.68), and sourcing it through valid prescriptions (ρ=0.50), although no single source was highly probable. This class had high proportions of self-assessed very good or excellent health while having generally lower proportions of mental health diagnoses. High-potency opioid use was more likely in this class than in the amphetamine self-medication or network-sourced for alertness classes.

#### Recreational Use

This class (23.9%) had a high probability of amphetamine use (ρ=1.00), with the highest probability of nonpharmaceutical stimulant use across classes (ρ=0.58). This class had high probabilities of nonmedically using stimulants for alertness (ρ=0.82) and for enjoyment or to get high (ρ=0.60). This class had moderate probabilities of both swallowing (ρ=0.70) and inhaling the medication (ρ=0.54), as well as primarily sourcing it through friends and family networks (ρ=0.70) and from dealers (ρ=0.53). This class also had higher proportions of prescription drug treatment in the last 12 months and nonmedical use of opioids of all potencies. This class had the highest proportions of anxiety disorders, major depressive disorders, and posttraumatic stress disorder. This group also had high proportions of nonpharmaceutical stimulant use, with approximately 1 in 3 using cocaine or methamphetamine in the last 12 months.

#### Nondiscriminatory Behaviors

This latent class (17.9%) showed high probabilities across most of the drugs, reasons, routes, and sources. This indicates that all these behaviors were prevalent in combination, but no single combination of behaviors particularly defined the class. This class consisted of a population that indiscriminately used prescription stimulants. This class had the highest likelihood of injecting prescription stimulants. It also had the highest proportion of drug treatment in the last 12 months (21%), while also having the highest proportion of self-assessed very good or excellent health (73%). In general, it had some of the highest proportions of mental health disorders. In this class, the proportion of nonpharmaceutical stimulant use ranged from 22% for MDMA to 28% for methamphetamine.

#### Demographic and Other Drug Use Characteristics

When looking at the demographic characteristics that differ among these 5 latent classes, 4 of them consisted of majority male individuals, whereas 1 (the network-sourced for alertness class) consisted of majority female individuals (61%). The amphetamine self-medication, nonamphetamine performance use, and nondiscriminatory behaviors classes in general had higher proportions of those who were married and had higher levels of education. The nonamphetamine performance use and nondiscriminatory behaviors classes had higher proportions of current or former armed forces personnel. There were no differences across classes regarding current student status, but there were differences in the proportions of health care professionals, with the highest proportions found in the nonamphetamine performance use and nondiscriminatory behaviors classes. The estimated mean age ranged from 32 to 34 years.

#### Association of Class Membership With DAST-10 Score and Drug Use Treatment

**Figure 2:**
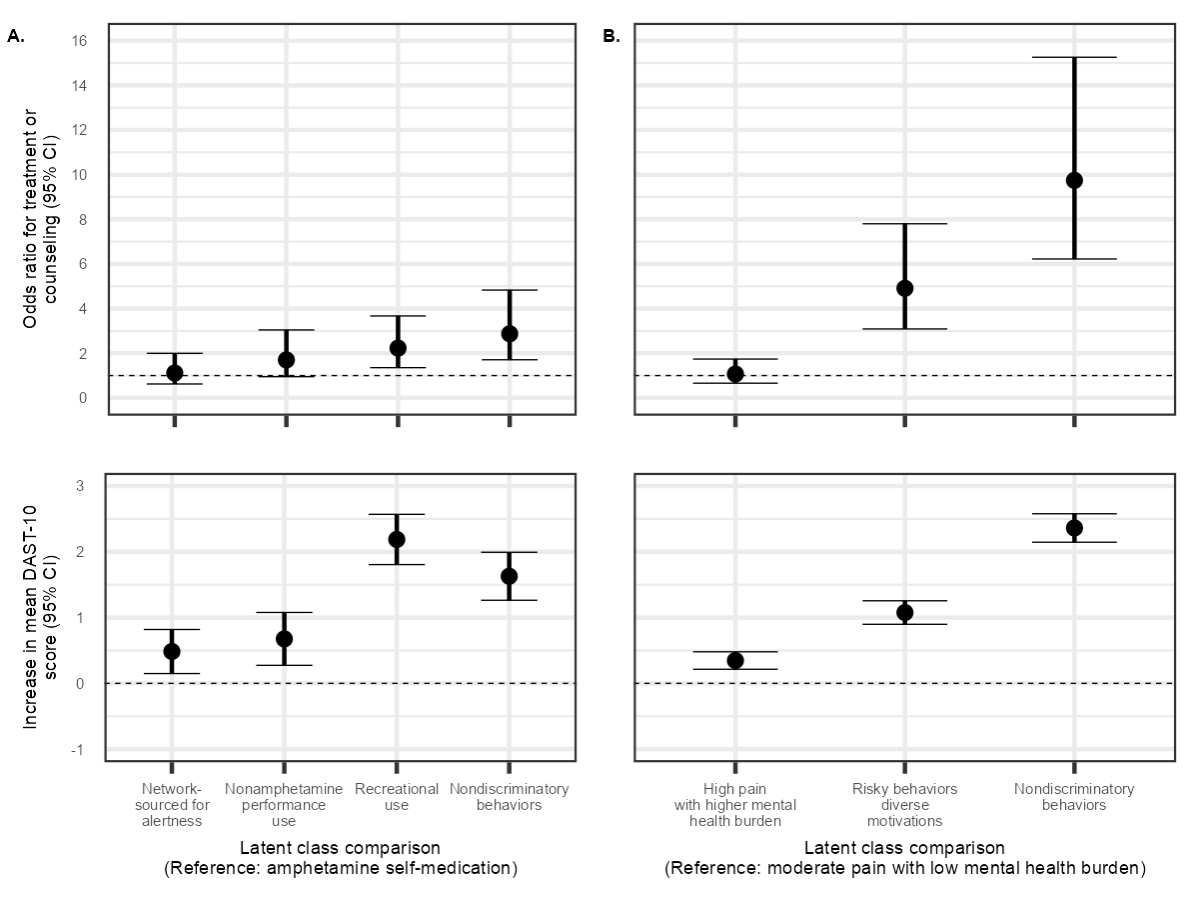
Latent class membership association with Drug Abuse Screening Test-10 (DAST-10) score and drug treatment history in the last 12 months. (A) Prescription stimulant nonmedical use. (B) Prescription opioid nonmedical use.

When modeling the association of class membership with DAST-10 score and drug use treatment in the last 12 months, the adjustment for confounders attenuated the effect estimates. Every class still had a statistically significant increase in mean DAST-10 score (*P*<.001) above the amphetamine self-medication class (Figure 2; Table S5 in Multimedia Appendix 1) after adjusting for confounding. The mean increase in estimates ranged from 0.48 DAST-10 points for the network-sourced stimulant for alertness class to 2.19 DAST-10 points for the recreational use class. The association of class membership with receiving treatment for the use of prescription or other illegal drugs in the last 12 months was elevated only for the recreational use class (odds ratio [OR] 2.23, 95% CI 1.35-3.67) and the nondiscriminatory behaviors class (OR 2.87, 95% CI 1.71-4.83) after adjusting for confounding compared with the amphetamine self-medication class.

### Subgroups of Opioid Nonmedical Use

#### Item Response and Model Fit Overview

Among adults who nonmedically used prescription opioids, medium-potency opioids were the most common drug (60.8%), followed by low-potency opioids (52.9%). The prevalence of use of nonpharmaceutical opioids was <6% each (Table S2 in Multimedia Appendix 1). The most common reasons for nonmedical use included to reduce pain (77.7%) and to relax or reduce stress or sleep (46.8%), whereas in the case of a little less than one-third of adults, the reasons included for enjoyment and to get high (28.1%). Receiving the prescription opioid through a valid prescription for oneself was the most common source (55%), followed by receiving from a friend or family member (49.9%).

**Figure 3:**
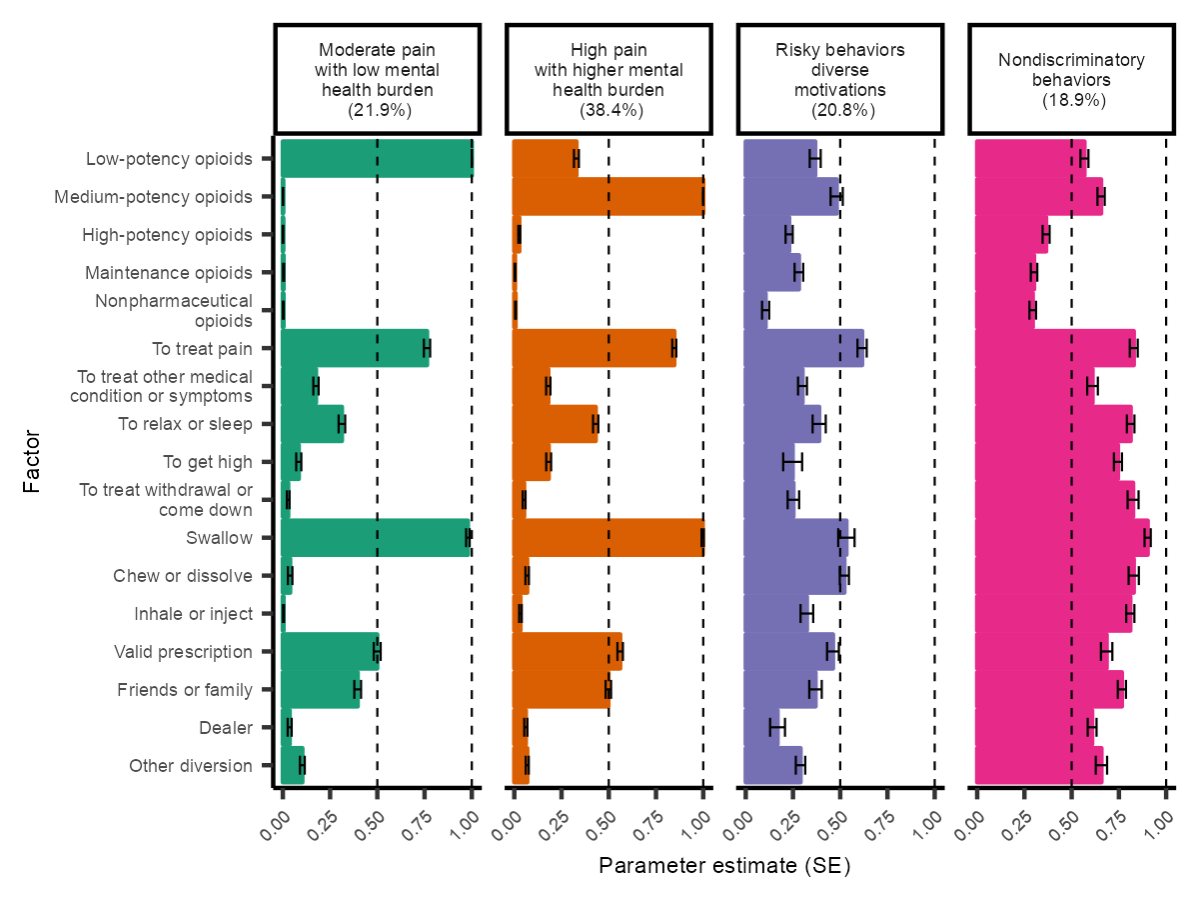
Opioid nonmedical use latent class item-response probabilities.

The opioid LCA resulted in up to 4 latent classes across converged models, and the 4-class model had the lowest BIC and appropriate fit statistics (Tables 1 and 2). Full item-response probabilities and 95% CI for each observed variable across the classes are included in Table S6 in Multimedia Appendix 1. Among adults nonmedically using prescription opioids, the interpretation of the 4 latent classes relied both on item-response probabilities and the posterior distributions of health factors to best understand the results. Their descriptions and prevalence estimates (Figure 3; Table S7 in Multimedia Appendix 1) are provided in the following subsections.

#### Moderate Pain With Low Mental Health Burden

This class (21.9%) was primarily defined by exclusive probability of the use of low-potency opioids (ρ=1.00), with zero probability of the use of other opioids. This class had a high probability of nonmedically using opioids to reduce pain (ρ=0.76); 51% had chronic or acute pain in the last 12 months, and 38% saw a physician for this pain. This class had a moderate probability of sourcing these low-potency opioids through a valid prescription (ρ=0.50) and through friends or family members (ρ=0.40). There was also a high probability of swallowing the medication (ρ=0.98), which is the intended route for most of these opioids. In comparison with the other classes, there were overall lower proportions of mental health diagnoses in this class.

#### High Pain With Higher Mental Health Burden

This class (38.4%) was primarily defined by medium-potency opioid use (ρ=1.00), with a moderate probability of low-potency opioid use (ρ=0.33). This class had the highest probability of nonmedically using opioids to reduce pain (ρ=0.85) and swallowing the medication (ρ=1.00). This class had the highest proportion of individuals who had experienced acute or chronic pain (60%), had seen a physician for this pain (44%), and had received a prescription opioid for this pain (34%). Similarly, this class had a moderate probability of sourcing opioids through a valid prescription (ρ=0.56) and through friends or family members (ρ=0.50). However, the proportions of anxiety disorders (38%) and major depressive disorder (15%) were elevated compared with other classes, considering that this class did not have the other risky drug-related behavior profiles.

#### Risky Behaviors With Diverse Motivations

This class (20.8%) was not defined by any 1 type of opioid use, meaning that all types, including maintenance drugs and nonpharmaceutical opioid use, were common among the class. This class had the lowest probability of nonmedically using opioids to reduce pain (ρ=0.62); reasons such as to prevent or treat withdrawal symptoms or a comedown from a high were elevated compared with the first 2 described classes. As there were diverse reasons for use, this indicates that the population was managing multiple drug effects beyond managing pain. This class showed approximately equal probabilities of some higher-risk behaviors such as other oral routes (chewing or dissolving medications, ρ=0.52) and nonoral routes (inhalation or injection, ρ=0.32). The use of stimulants was higher for this class than for the pain-oriented classes. This class had the lowest percentage of individuals who had experienced acute or chronic pain (45%). Compared with the other classes, there were higher proportions of several mental health disorders, including autism spectrum disorder and bipolar disorder. Notably, this class began to show higher nonpharmaceutical opioid use, including heroin (8%) and fentanyl (5%) use.

#### Nondiscriminatory Behaviors

As in the case of the stimulant analysis, a latent class (18.9%) appeared that had high probabilities across most of the drugs, reasons, routes, and sources. This indicates that all these behaviors were prevalent in combination, but no single combination of behaviors particularly defined the class. This class consisted of a population that indiscriminately used opioids. This class had the highest likelihood of injecting prescription opioids and nonmedically using stimulants. This class also had the highest proportions (at most 2-fold higher) of mental health disorders across classes, and 55% had experienced acute or chronic pain in the last year. Notably, there was 21% heroin use and 18% fentanyl use in the last 12 months.

#### Demographic and Other Drug Use Characteristics

When looking at the demographic characteristics that differ among these 4 latent classes, the risky behaviors with diverse motivations and nondiscriminatory behaviors classes consisted of majority male individuals, whereas the other 2 classes consisted of approximately equal numbers of male individuals and female individuals. The nondiscriminatory behaviors class in general had higher proportions of those who had advanced education and were health care professionals, current students, or current or former armed forces personnel. There were no differences across classes across income, marital status, or most race categories. The mean age ranged from 33 to 41 years.

#### Association of Class Membership With DAST-10 Score and Drug Use Treatment

When modeling the association of class membership with DAST-10 score and drug use treatment in the last 12 months, the adjustment for confounders attenuated the effect estimates. Every class still had a statistically significant increase in mean DAST-10 score (*P*<.001) above the moderate pain with low mental health burden class (Figure 2; Table S8 in Multimedia Appendix 1) after adjusting for confounding. These mean increase in estimates ranged from 0.35 DAST-10 points for the high pain with higher mental health burden class to 2.36 DAST-10 points for the nondiscriminatory behaviors class. The association of class membership with receiving treatment for the use of prescription or other illegal drugs in the last 12 months were elevated for the risky behaviors with diverse motivations (OR 4.91, 95% CI 3.09-7.80) and nondiscriminatory behaviors (OR 9.74, 95% CI 6.22-15.26) classes after adjusting for confounding compared with the moderate pain with low mental health burden class.

## Discussion

### Principal Findings

Overall, among adults in the United States, the prevalence of prescription stimulant nonmedical use was lower than that for prescription opioids, with amphetamine having the highest prevalence at 1.9% and methylphenidate and modafinil having similar prevalence estimates of 0.2% to 0.3%. Both prescription stimulant and opioid nonmedical use populations were heterogeneous, with distinct behavioral patterns and associated demographic, mental health, and risk factor profiles. This is consistent with other latent profile work demonstrating that drug use can peak at many different points throughout adulthood, and peaks are associated with a multitude of prior factors [17]. Among adults who nonmedically used prescription stimulants, 5 distinct latent classes emerged, ranging in prevalence rates from 14% to 26%, indicating that there is no one dominant use pattern. Among adults who nonmedically used prescription opioids, 4 distinct latent classes emerged, where the high pain with higher mental health burden class was twice as common as the other classes. For each drug category, the identity of the drug used was a factor that differentiated classes, but other behavioral characteristics were important to the class definitions as well.

Unsurprisingly, amphetamine use largely separated from other prescription stimulant use, which is consistent with dispensing volume. The amphetamine self-medication class largely used amphetamine to treat medical symptoms and therefore could represent a group of individuals with untreated or undertreated ADHD because less than half of the subgroup reported an ADHD diagnosis. The appearance of this latent class was expected among the general population of adults, where ADHD diagnoses are challenging [29]. However, our analysis identified 2 latent classes of stimulant users that are unique contributions to our understanding of those who use prescription stimulants. First, we identified the network-sourced for alertness latent class with a prevalence of 20%. Although the behavioral pattern seems similar to behavioral patterns documented among college-aged students [30], we demonstrated that this type of behavioral pattern is not confined to college students or young adults. This latent class consisted of predominately female individuals (61%), had lower diagnoses of ADHD, and obtained their medications almost exclusively through friends or family. This could be evidence pointing toward some female individuals with underdiagnoses of ADHD self-medicating, with other behaviors being low risk, such as mostly swallowing their medication and low report of use to get high. Prior work has shown that female individuals may have been a clinically underdiagnosed population for ADHD, given the differences in clinical symptomology between male individuals and female individuals, particularly with lower rates of diagnoses in childhood [29]. Second, this is the first study to identify a subgroup of prescription stimulant users who seem to be largely using the stimulants for performance reasons but are predominately using nonamphetamine drugs such as methylphenidate and modafinil. This group, although the least common, seems to also have a higher-risk behavioral profile, including routes that involve tampering, such as inhalation or chewing of medications, and sourcing through diversion, potentially exposing them to adulterated drug products. Both classes may need diverse intervention strategies to address their health risks.

Both prescription stimulant and opioid LCAs in this study identified a similar class, the nondiscriminatory behaviors class, which was characterized by a high probability of the use of multiple substances and risky behaviors, particularly injection of the drug and nonmedical use across drug classes. These classes were also characterized by increased proportions of mental health disorders and previous substance use treatment. This finding is consistent with the findings of 2 other LCAs of prescription stimulants [31,32] and a systematic review of latent classes among opioid users [13]. In addition, there is also evidence that similar classes of behavioral patterns are present among adolescents who nonmedically use prescription stimulants [31], potentially indicating that, for some, these use patterns may begin early when health care providers are more likely to interface with adolescents, allowing for opportunities for education.

A systematic review of other stimulant use studies found that the acquisition of prescription stimulants from friends and family networks [8] ranged from 50% to 90%, and this study found that 55% of the respondents reported receiving the medication from a friend or family member. Interestingly, some of the distinct latent classes were almost exclusively obtaining their medicines through these networks. The network-sourced for alertness, recreational use, and nondiscriminatory behaviors subgroups (accounting for 60% of this population) are particularly concerning because these classes tended to not obtain the drug from a health care professional and therefore are potentially less exposed to information guiding proper dosage, risks of co-use with other drugs, contraindications to stimulant use, and other safe medication practices typically communicated through health care professionals. Furthermore, interventions primarily implemented through a health care professional would have limited direct impact on these groups. Future interventions may need to target specific subgroups; for instance, the increased proportion of health care professionals among several of the stimulant use classes can be presumed to be aware of the risks and may warrant a different approach than more traditional risk education.

The opioid LCA showed more variance among mental health disorders and substance use treatment than the stimulant population. Generally, the 4 latent classes identified among those who have nonmedically used a prescription opioid were largely distinguished by type of opioid use, pain history, and mental health burden. These findings were generally consistent with those of a systematic review of all LCAs among persons who use opioids and do indicate that there is a subgroup of opioid users at risk who are not necessarily at the level of requiring treatment but should be considered for interventions to prevent progression to opioid use disorders [14]. However, the findings presented here go beyond past works, which have focused on polysubstance use and transition behaviors [13,14,33], to include a series of behaviors that better represent a person-centric perspective on factors that compose drug-related behaviors in adults.

A precision public health approach [18] aimed at preventing progression to more severe drug-related outcomes would address the differences in subgroups who nonmedically use stimulants. The most common providers of prescription stimulants include the 3 specialties of psychiatry, pediatrics, and primary care physicians [4]. For the amphetamine self-medication class, the lack of high-risk behaviors could mean that an intervention with this subgroup would focus more on treating unrecognized mental health conditions rather than high-risk consequences, whereas an intervention with other latent classes such as the recreational use and nondiscriminatory behaviors classes, which show adjusted DAST-10 scores on average almost 2 points higher, would likely involve evaluating substance use disorders. A next step in studying prescription stimulant nonmedical use could be to connect the subgroup behaviors to the resulting consequences and estimate the effectiveness of potential interventions.

### Strengths and Limitations

This study has several notable strengths. First, the NMURx program sampling was not related to drug use, and the adults in the sample more likely represent the full range of stimulant subgroups to study, whereas many other studies focus on more specialized populations with less generalizable results. Second, the large sample size and the inclusion of behaviors in addition to specific substances in our latent models allowed us to distinguish more nuanced behavioral patterns among prescription stimulant nonmedical users, identifying 2 classes in the general population that are underrepresented in research. One limitation to this study is the *drug-centric* approach, focusing on separate stimulant and opioid profiles. Future research would benefit from performing similar analyses considering all drug use profiles and exploring more polysubstance use behaviors to further our understanding of the evolving drug use landscape.

### Conclusion

To increase the effectiveness of drug-related health interventions, the diversity in how and why people use drugs must be considered. A more precise understanding of how behaviors tend to co-occur would improve efficacy and efficiency in deploying resources to support the overall health of those who use drugs, and it would improve how we communicate with, and connect to, those at risk for severe drug outcomes.

## Appendix

Multimedia Appendix 1 Additional results.

Multimedia Appendix 2 Consent language.

## Abbreviations

ADHD: attention-deficit/hyperactivity disorder
AIC: Akaike information criterion
BIC: Bayesian information criterion
DAST-10: Drug Abuse Screening Test-10
LCA: latent class analysis
MDMA: 3,4-methylenedioxy-methamphetamine
NMURx: Nonmedical Use of Prescription Drugs
OR: odds ratio

## References

[1] Simon V, Czobor P, Bálint S, Mészáros A, Bitter I (2009). Prevalence and correlates of adult attention-deficit hyperactivity disorder: meta-analysis. Br J Psychiatry.

[2] Banerjee D, Vitiello MV, Grunstein RR (2004). Pharmacotherapy for excessive daytime sleepiness. Sleep Med Rev.

[3] Zablotsky B, Black LI (2020). Prevalence of children aged 3-17 years with developmental disabilities, by urbanicity: United States, 2015-2018. Natl Health Stat Report.

[4] Board AR, Guy G, Jones CM, Hoots B (2020). Trends in stimulant dispensing by age, sex, state of residence, and prescriber specialty - United States, 2014-2019. Drug Alcohol Depend.

[5] Katzman MA, Bilkey TS, Chokka PR, Fallu A, Klassen LJ (2017). Adult ADHD and comorbid disorders: clinical implications of a dimensional approach. BMC Psychiatry.

[6] 2020 National Survey of Drug Use and Health (NSDUH) releases. Substance Abuse and Mental Health Services Administration.

[7] Dart RC (2009). Monitoring risk: post marketing surveillance and signal detection. Drug Alcohol Depend.

[8] Faraone SV, Rostain AL, Montano CB, Mason O, Antshel KM, Newcorn JH (2020). Systematic review: nonmedical use of prescription stimulants: risk factors, outcomes, and risk reduction strategies. J Am Acad Child Adolesc Psychiatry.

[9] McCabe SE, West BT (2013). Medical and nonmedical use of prescription stimulants: results from a national multicohort study. J Am Acad Child Adolesc Psychiatry.

[10] Sweeney CT, Sembower MA, Ertischek MD, Shiffman S, Schnoll SH (2013). Nonmedical use of prescription ADHD stimulants and preexisting patterns of drug abuse. J Addict Dis.

[11] Mattson CL, Tanz LJ, Quinn K, Kariisa M, Patel P, Davis NL (2021). Trends and geographic patterns in drug and synthetic opioid overdose deaths - United States, 2013-2019. MMWR Morb Mortal Wkly Rep.

[12] Black JC, Bau GE, Iwanicki JL, Dart RC (2021). Association of medical stimulants with mortality in the US from 2010 to 2017. JAMA Intern Med.

[13] Karamouzian M, Pilarinos A, Hayashi K, Buxton JA, Kerr T (2022). Latent patterns of polysubstance use among people who use opioids: a systematic review. Int J Drug Policy.

[14] de Jonge MC, Bukman AJ, van Leeuwen L, Onrust SA, Kleinjan M (2022). Latent classes of substance use in young adults - a systematic review. Subst Use Misuse.

[15] Lopez-Quintero C, Pérez de los Cobos J, Hasin DS, Okuda M, Wang S, Grant BF, Blanco C (2011). Probability and predictors of transition from first use to dependence on nicotine, alcohol, cannabis, and cocaine: results of the National Epidemiologic Survey on Alcohol and Related Conditions (NESARC). Drug Alcohol Depend.

[16] McCabe SE, Schulenberg JE, O'Malley PM, Patrick ME, Kloska DD (2014). Non-medical use of prescription opioids during the transition to adulthood: a multi-cohort national longitudinal study. Addiction.

[17] McCabe SE, Schulenberg JE, Schepis TS, Evans-Polce RJ, Wilens TE, McCabe VV, Veliz PT (2022). Trajectories of prescription drug misuse among US adults from ages 18 to 50 years. JAMA Netw Open.

[18] Khoury MJ, Iademarco MF, Riley WT (2016). Precision public health for the era of precision medicine. Am J Prev Med.

[19] Black JC, Rockhill K, Forber A, Amioka E, May KP, Haynes CM, Dasgupta N, Dart RC (2019). An online survey for pharmacoepidemiological investigation (survey of non-medical use of prescription drugs program): validation study. J Med Internet Res.

[20] Black JC, Forber A, Severtson SG, Rockhill K, May KP, Amioka E, Schwarz J, Iwanicki J, Dart RC (2021). Drug product dispensing and estimates of use in a general population survey as a signal detection problem. Pharmacoepidemiol Drug Saf.

[21] Dowell D, Haegerich TM, Chou R (2016). CDC guideline for prescribing opioids for chronic pain--United States, 2016. JAMA.

[22] Skinner HA (1982). The drug abuse screening test. Addict Behav.

[23] Yudko E, Lozhkina O, Fouts A (2007). A comprehensive review of the psychometric properties of the Drug Abuse Screening Test. J Subst Abuse Treat.

[24] Collins LM, Lanza ST (2010). Latent Class and Latent Transition Analysis: With Applications in the Social, Behavioral, and Health Sciences.

[25] Weller BE, Bowen NK, Faubert SJ (2020). Latent class analysis: a guide to best practice. J Black Psychol.

[26] Bakk Z, Kuha J (2021). Relating latent class membership to external variables: an overview. Br J Math Stat Psychol.

[27] Nagin D (2005). Group-based Modeling of Development.

[28] Lanza ST, Collins LM, Lemmon DR, Schafer JL (2007). PROC LCA: a SAS procedure for Latent class analysis. Struct Equ Modeling.

[29] Katzman MA, Bilkey T, Chokka PR, Fallu A, Klassen LJ (2016). Re: is adult attention-deficit hyperactivity disorder being overdiagnosed?. Can J Psychiatry.

[30] Teter CJ, McCabe SE, LaGrange K, Cranford JA, Boyd CJ (2006). Illicit use of specific prescription stimulants among college students: prevalence, motives, and routes of administration. Pharmacotherapy.

[31] Chen L-Y, Crum RM, Martins SS, Kaufmann CN, Strain EC, Mojtabai R (2014). Patterns of concurrent substance use among nonmedical ADHD stimulant users: results from the National Survey on Drug Use and Health. Drug Alcohol Depend.

[32] Timko C, Han X, Woodhead E, Shelley A, Cucciare MA (2018). Polysubstance use by stimulant users: health outcomes over three years. J Stud Alcohol Drugs.

[33] Barton AW, Reinhart CA, Campbell CC, Smith DC, Albarracin D (2021). Opioid use at the transition to emerging adulthood: a latent class analysis of non-medical use of prescription opioids and heroin use. Addict Behav.

