# Supplementary material for: Differing Behaviors Around Adult Nonmedical Use of Prescription Stimulants and Opioids: Latent Class Analysis
Source: J Med Internet Res. 2023 Sep 20;25:e46742. doi: 10.2196/46742 (PMC10551786; doi:10.2196/46742)
Supplement: Multimedia Appendix 1 [file jmir_v25i1e46742_app1.docx]

Multimedia Appendix 1: Additional Results

**Table S1: Demographic and Health Characteristics**

| **Characteristic** | | **Total Population**  **% (95% CI)** | **Prescription Stimulant Non-Medical Use**  **% (95% CI)** | **Prescription Opioid Non-Medical Use**  **% (95% CI)** |
| --- | --- | --- | --- | --- |
| **Demographics** | |  |  |  |
| Age (years), Mean (95% CI) | | 47.0 (46.9, 47.1) | 32.9 (32.3, 33.5) | 38.7 (38.2, 39.1) |
| Male | | 48.7 (48.3, 49.0) | 58.5 (55.9, 61.2) | 57.5 (56.0, 59.0) |
| Census Region | |  |  |  |
|  | Northeast | 17.6 (17.3, 17.9) | 15.6 (13.8, 17.4) | 14.5 (13.5, 15.5) |
|  | Midwest | 20.9 (20.6, 21.2) | 17.3 (15.3, 19.2) | 17.8 (16.6, 18.9) |
|  | South | 37.9 (37.5, 38.2) | 40.5 (37.9, 43.1) | 41.8 (40.3, 43.3) |
|  | West | 23.7 (23.4, 24.0) | 26.7 (24.3, 29.1) | 26.0 (24.6, 27.3) |
| Race^a^ | |  |  |  |
|  | American Indian/Alaska Native | 1.8 (1.7, 1.9) | 2.7 (1.8, 3.6) | 2.5 (2.0, 2.9) |
|  | Asian | 5.7 (5.5, 5.9) | 6.3 (4.9, 7.6) | 5.5 (4.7, 6.2) |
|  | Black or African American | 10.2 (9.9, 10.4) | 11.4 (9.6, 13.2) | 16.2 (15.0, 17.4) |
|  | Native Hawaiian/Pacific Islander | 0.4 (0.4, 0.5) | 0.7 (0.2, 1.2) | 0.8 (0.5, 1.1) |
|  | White | 81.1 (80.7, 81.4) | 78.2 (75.8, 80.5) | 73.3 (71.9, 74.8) |
|  | Other Race | 3.6 (3.4, 3.7) | 4.3 (3.1, 5.5) | 4.8 (4.1, 5.5) |
| Ethnicity | |  |  |  |
|  | Hispanic/Latino | 9.0 (8.7, 9.2) | 16.7 (14.7, 18.7) | 15.1 (14.0, 16.2) |
|  | Not Hispanic/Latino | 91.0 (90.8, 91.3) | 83.3 (81.3, 85.3) | 84.9 (83.8, 86.0) |
| Household Income | |  |  |  |
|  | <$50,000 | 44.4 (44.0, 44.8) | 43.8 (41.1, 46.4) | 47.7 (46.1, 49.2) |
|  | $50,000 - $74,999 | 37.1 (36.7, 37.4) | 38.2 (35.6, 40.7) | 36.9 (35.4, 38.4) |
|  | >$75,000 | 18.5 (18.2, 18.8) | 18.1 (16.1, 20.0) | 15.4 (14.4, 16.5) |
| Marital Status | |  |  |  |
|  | Married | 52.1 (51.8, 52.5) | 41.0 (38.4, 43.5) | 47.6 (46.1, 49.1) |
|  | Never Married | 30.5 (30.1, 30.8) | 46.0 (43.3, 48.6) | 35.5 (34.0, 37.0) |
|  | Widowed/Divorced/Separated | 17.4 (17.2, 17.7) | 13.1 (11.5, 14.7) | 16.9 (15.8, 18.0) |
| Highest Level of Education | |  |  |  |
|  | High School or Less | 20.6 (20.3, 20.9) | 19.6 (17.5, 21.8) | 23.6 (22.3, 24.9) |
|  | Some College or Associates Degree | 32.6 (32.2, 32.9) | 34.7 (32.1, 37.2) | 35.2 (33.7, 36.6) |
|  | Bachelor’s Degree or Trade School | 32.0 (31.6, 32.3) | 28.2 (25.8, 30.6) | 27.9 (26.6, 29.3) |
|  | Graduate Degree | 14.8 (14.6, 15.1) | 17.5 (15.6, 19.3) | 13.3 (12.4, 14.3) |
| Current Healthcare Professional | | 4.6 (4.4, 4.8) | 10.9 (9.4, 12.4) | 8.1 (7.3, 8.9) |
| Current Student | | 7.5 (7.3, 7.7) | 18.5 (16.2, 20.7) | 11.7 (10.6, 12.8) |
| Current or Former Armed Forces | | 8.8 (8.6, 9.0) | 7.7 (6.5, 9.0) | 8.2 (7.4, 8.9) |
| **Health Factors in the Last 12 Months** | | |  |  |
| Private Health Insurance Coverage | | 65.1 (64.7, 65.4) | 57.3 (54.7, 59.9) | 57.8 (56.3, 59.3) |
| Tobacco Use | | 13.8 (13.6, 14.0) | 46.6 (44.1, 49.2) | 39.1 (37.8, 40.5) |
| Alcohol Use | | 68.1 (67.6, 68.6) | 60.2 (57.1, 63.3) | 62.9 (61.1, 64.7) |
| General Health Self-Assessment | |  |  |  |
|  | Poor/Fair | 13.0 (12.8, 13.3) | 11.4 (9.8, 13.0) | 16.3 (15.2, 17.3) |
|  | Good | 37.6 (37.2, 37.9) | 33.7 (31.2, 36.1) | 37.2 (35.7, 38.7) |
|  | Very Good/Excellent | 49.4 (49.0, 49.8) | 54.9 (52.3, 57.6) | 46.5 (45.0, 48.0) |
| Hospital Stay Overnight | | 9.6 (9.4, 9.8) | 18.2 (16.4, 20.1) | 19.7 (18.6, 20.9) |
| Received Treatment/Counseling | |  |  |  |
|  | Alcohol Use | 1.3 (1.2, 1.3) | 8.1 (6.9, 9.4) | 5.4 (4.8, 5.9) |
|  | Prescription or Illicit Drug Use | 1.5 (1.5, 1.6) | 12.1 (10.6, 13.6) | 9.1 (8.3, 9.9) |
| Pain Experience | |  |  |  |
|  | Acute or Chronic Pain | 36.1 (35.7, 36.4) | 45.3 (42.7, 47.9) | 54.2 (52.7, 55.7) |
|  | Acute or Chronic Pain & Saw Doctor | 23.1 (22.8, 23.4) | 27.5 (25.3, 29.8) | 38.0 (36.5, 39.4) |
|  | Acute or Chronic Pain & Received Pain Reliever Prescription | 13.7 (13.5, 14.0) | 19.7 (17.7, 21.6) | 29.2 (27.9, 30.5) |
| Mental Health Diagnoses | |  |  |  |
|  | ADHD/ADD | 5.1 (5.0, 5.3) | 29.4 (27.1, 31.8) | 12.5 (11.5, 13.5) |
|  | Any Anxiety Disorder | 20.4 (20.1, 20.7) | 41.9 (39.3, 44.5) | 34.8 (33.4, 36.2) |
|  | Autism Spectrum Disorder | 1.2 (1.1, 1.3) | 4.3 (3.2, 5.3) | 2.6 (2.1, 3.1) |
|  | Bipolar Disorder | 4.0 (3.9, 4.1) | 14.9 (13.2, 16.6) | 12.1 (11.2, 13.0) |
|  | Borderline Personality Disorder | 1.0 (1.0, 1.1) | 4.6 (3.7, 5.6) | 3.6 (3.1, 4.2) |
|  | Eating Disorder | 1.5 (1.4, 1.6) | 5.3 (4.2, 6.5) | 4.4 (3.8, 5.0) |
|  | Major Depressive Disorder | 8.2 (8.0, 8.4) | 17.7 (15.8, 19.7) | 14.0 (13.0, 15.0) |
|  | Post-Traumatic Stress Disorder | 4.5 (4.4, 4.6) | 12.0 (10.4, 13.7) | 10.0 (9.1, 10.8) |
|  | Schizophrenia | 0.7 (0.7, 0.8) | 2.6 (1.8, 3.4) | 1.9 (1.5, 2.3) |
| DAST-10 Score, Mean (95% CI) | | 0.6 (0.6, 0.6) | 2.8 (2.7, 2.9) | 2.1 (2.0, 2.2) |

All data presented as % (95% Confidence Interval) unless otherwise noted

^a^ Respondents may select more than one race

**Table S2: Behavioral Endorsement by Drug Use**

| **Drug Use Behavior** | | **Prescription Stimulant Non-Medical Use**  **% (95% CI)** | **Prescription Opioid Non-Medical Use**  **% (95% CI)** |
| --- | --- | --- | --- |
| Non-Medical Use of Prescription Drug | |  |  |
|  | Amphetamine | 79.9 (77.9, 81.9) | NA |
|  | Methylphenidate | 17.7 (15.8, 19.6) | NA |
|  | Modafinil | 12.5 (11.0, 14.1) | NA |
|  | Low-Potency Opioids | NA | 52.9 (51.4, 54.4) |
|  | Medium-Potency Opioids | NA | 60.8 (59.3, 62.3) |
|  | High-Potency Opioids | NA | 12.7 (11.7, 13.6) |
|  | Maintenance Opioids | NA | 11.8 (10.8, 12.7) |
| Recreational Drug Use | |  |  |
|  | Any Recreational Stimulant | 35.3 (32.8, 37.7) | NA |
|  | Cocaine Powder | 19.0 (16.9, 21.0) | NA |
|  | Crack Cocaine | 7.1 (5.9, 8.3) | NA |
|  | Illicit Amphetamine | 9.3 (7.9, 10.6) | NA |
|  | MDMA | 13.1 (11.3, 14.8) | NA |
|  | Methamphetamine | 16.1 (14.4, 17.9) | NA |
|  | Any Recreational Opioid | NA | 8.0 (7.3, 8.8) |
|  | Heroin | NA | 5.8 (5.1, 6.4) |
|  | Illicit Fentanyl | NA | 4.6 (4.0, 5.1) |
| Reason for Prescription Non-Medical Use | |  |  |
|  | To Treat a Medical Condition or Symptom | 35.0 (32.6, 37.5) | 28.5 (27.2, 29.9) |
|  | To Hurt Yourself or End Life | 11.4 (9.9, 12.8) | 8.8 (8.0, 9.6) |
|  | For Enjoyment or To Get High | 35.2 (32.8, 37.7) | 28.1 (26.7, 29.4) |
|  | To Stay Awake or Be Alert | 59.6 (57.0, 62.2) | NA |
|  | To Focus or Get Work Done | 62.8 (60.3, 65.4) | NA |
|  | To Increase Athletic Performance | 19.4 (17.4, 21.4) | NA |
|  | For Weight Loss or To Control Appetite | 26.6 (24.3, 28.8) | NA |
|  | To Reduce Pain | NA | 77.7 (76.4, 79.0) |
|  | To Relax/Reduce Stress or Sleep | NA | 46.8 (45.3, 48.3) |
|  | To Prevent or Treat Withdrawal Symptoms | NA | 18.9 (17.7, 20.0) |
|  | To Come Down from a High or Another Drug | NA | 13.0 (12.0, 13.9) |
| Route of Administration for Prescription Non-Medical Use | | |  |
|  | Swallowed | 83.1 (81.2, 85.0) | 87.9 (86.9, 88.8) |
|  | Other Oral | 35.2 (32.8, 37.7) | 30.0 (28.6, 31.3) |
|  | Inhaled | 28.5 (26.2, 30.8) | 16.9 (15.8, 17.9) |
|  | Injected | 16.5 (14.7, 18.2) | 15.0 (13.9, 16.0) |
| Source of Drug for Prescription Non-Medical Use | | | |
|  | Valid Prescription for Yourself | 41.5 (38.9, 44.1) | 55.0 (53.5, 56.5) |
|  | Friend or Family Member | 55.4 (52.8, 58.1) | 49.9 (48.4, 51.4) |
|  | Dealer | 25.0 (22.7, 27.2) | 18.1 (17.0, 19.2) |
|  | Prescription Diversion | 20.9 (19.0, 22.9) | 20.0 (18.8, 21.2) |
|  | Other Non-Prescription Diversion | 21.2 (19.3, 23.2) | 16.8 (15.7, 17.9) |

NA indicates behavior was not assessed for that drug user group

**Table S3: Latent Class Modeling Results – Non-Medical Use of Prescription Stimulants**

| **Model Parameters** | | **Amphetamine Self-Medication** | **Network Sourced for Alertness** | **Non-Amphetamine Performance Use** | **Recreational Use** | **Non-Discriminatory Behaviors** |
| --- | --- | --- | --- | --- | --- | --- |
| Prevalence | | 0.26 (0.02) | 0.19 (0.01) | 0.14 (0.01) | 0.24 (0.02) | 0.18 (0.01) |
| Drug Use | |  |  |  |  |  |
|  | Amphetamine NMU | **1.00 (0.00)** | 0.08 (0.04) | **1.00 (0.00)** | **1.00 (0.00)** | **0.58 (0.04)** |
|  | Methylphenidate NMU | 0.04 (0.01) | **0.55 (0.04)** | 0.02 (0.01) | 0.09 (0.02) | 0.37 (0.03) |
|  | Modafinil NMU | 0.02 (0.01) | **0.49 (0.04)** | 0.02 (0.01) | 0.00 (0.00) | 0.29 (0.03) |
|  | Any Recreational Stimulant Use | 0.08 (0.02) | 0.27 (0.04) | 0.28 (0.03) | **0.58 (0.03)** | **0.57 (0.03)** |
| Reason for Prescription NMU | |  |  |  |  |  |
|  | Medical Condition or Symptom | 0.49 (0.03) | 0.38 (0.04) | 0.06 (0.02) | 0.14 (0.03) | **0.72 (0.03)** |
|  | Stay Awake/Focus | **0.65 (0.03)** | **0.70 (0.03)** | **0.89 (0.03)** | **0.82 (0.02)** | **0.89 (0.02)** |
|  | Enjoyment/To Get High | 0.09 (0.02) | 0.18 (0.04) | 0.15 (0.03) | **0.60 (0.03)** | **0.75 (0.03)** |
| Route of Administration for Prescription NMU | |  |  |  |  |  |
|  | Swallowed | **0.92 (0.02)** | **0.68 (0.04)** | **1.00 (0.00)** | **0.70 (0.03)** | **0.81 (0.03)** |
|  | Other Oral | 0.18 (0.03) | 0.40 (0.04) | 0.05 (0.02) | 0.36 (0.03) | **0.88 (0.03)** |
|  | Inhaled | 0.03 (0.01) | 0.17 (0.04) | 0.00 (0.00) | **0.54 (0.04)** | **0.71 (0.03)** |
|  | Injected | 0.01 (0.01) | 0.11 (0.03) | 0.00 (0.00) | 0.07 (0.01) | **0.73 (0.04)** |
| Source of Drug for Prescription NMU | |  |  |  |  |  |
|  | Valid Prescription | **0.76 (0.03)** | **0.50 (0.04)** | 0.04 (0.02) | 0.12 (0.03) | **0.66 (0.04)** |
|  | Friend or Family Member | 0.17 (0.04) | 0.27 (0.04) | **0.99 (0.01)** | **0.70 (0.03)** | **0.67 (0.03)** |
|  | Dealer | 0.04 (0.02) | 0.14 (0.03) | 0.00 (0.00) | **0.53 (0.03)** | **0.53 (0.03)** |
|  | Other Diversion | 0.18 (0.02) | 0.45 (0.05) | 0.02 (0.01) | 0.09 (0.02) | **0.90 (0.03)** |

Data shown as probability (standard error); item-response probabilities >0.5 bolded facilitate interpretation

**Table S4: Characteristics among Non-Medical Use of Prescription Stimulants Latent Classes**

| **Characteristic** | | **Amphetamine Self-Medication** | **Network Sourced for Alertness** | **Non-Amphetamine Performance Use** | **Recreational Use** | **Non-Discriminatory Behaviors** | **Holm P-value** |
| --- | --- | --- | --- | --- | --- | --- | --- |
| Unweighted N | | 477 | 336 | 320 | 478 | 472 | **-** |
| **Demographics** | | | | | | | |
| Age (years), Mean (95% CI) | | 34.2 (32.9, 35.6) | 32.2 (30.8, 33.7) | 33.6 (32.0, 35.2) | 31.6 (30.4, 32.7) | 33.2 (32.1, 34.2) | <.001 |
| Sex: Male, % (95% CI) | | 52.9 (47.4, 58.3) | 39.1 (33.0, 45.2) | 63.9 (57.3, 70.5) | 64.9 (59.8, 70.0) | 76.9 (72.3, 81.5) | <.001 |
| Race^a^, % (95% CI) | |  |  |  |  |  |  |
|  | American Indian or Alaska Native | 2.1 (0.8, 3.5) | 1.6 (0.4, 2.9) | 2.1 (0.1, 4.0) | 4.1 (1.6, 6.5) | 3.7 (1.0, 6.3) | >.99 |
|  | Asian | 6.6 (3.7, 9.5) | 3.9 (1.5, 6.4) | 8.2 (4.6, 11.9) | 7.5 (4.0, 11.0) | 5.4 (2.9, 8.0) | >.99 |
|  | Black or African American | 15.4 (11.0, 19.8) | 8.3 (4.9, 11.7) | 12.2 (7.4, 17.1) | 6.3 (3.4, 9.1) | 14.9 (10.7, 19.1) | .02 |
|  | Native Hawaiian or Pacific Islander | Suppressed | Suppressed | 1.1 (0.0, 2.2) | Suppressed | Suppressed | >0.99 |
|  | White | 73.6 (68.4, 78.7) | 83.6 (78.7, 88.5) | 73.4 (67.1, 79.7) | 84.3 (80.1, 88.5) | 74.1 (68.9, 79.3) | .01 |
|  | Other Race | 5.0 (2.5, 7.5) | 4.9 (1.7, 8.0) | 5.6 (1.7, 9.4) | 2.7 (1.3, 4.0) | 4.1 (1.2, 6.9) | >0.99 |
| Ethnicity, % (95% CI) | |  |  |  |  |  |  |
|  | Hispanic or Latinx | 18.2 (13.9, 22.6) | 15.9 (11.1, 20.7) | 18.4 (13.0, 23.7) | 9.1 (6.1, 12.0) | 24.1 (19.1, 29.1) | .001 |
| Annual Household Income, % (95% CI) | |  |  |  |  |  | <.001 |
|  | <$50,000 | 49.4 (44.0, 54.9) | 45.5 (39.1, 51.8) | 36.0 (29.0, 42.9) | 51.7 (46.2, 57.2) | 29.3 (24.0, 34.6) |  |
|  | $50,000 - $74,999 | 37.4 (32.0, 42.7) | 35.1 (29.2, 41.0) | 43.5 (36.8, 50.2) | 35.0 (29.8, 40.2) | 42.9 (37.5, 48.3) |  |
|  | >$75,000 | 13.2 (9.6, 16.8) | 19.4 (14.5, 24.4) | 20.6 (15.5, 25.7) | 13.3 (9.2, 17.4) | 27.8 (23.1, 32.5) |  |
| Marital Status, % (95% CI) | |  |  |  |  |  | <.001 |
|  | Married | 39.7 (34.5, 45.0) | 33.2 (27.4, 39.0) | 53.9 (46.9, 60.8) | 24.5 (20.2, 28.7) | 63.3 (57.8, 68.7) |  |
|  | Never Married | 12.6 (9.4, 15.7) | 13.7 (9.8, 17.6) | 9.8 (6.2, 13.4) | 17.2 (13.5, 20.9) | 10.1 (6.8, 13.4) |  |
|  | Widowed or Divorced or Separated | 47.7 (42.2, 53.3) | 53.1 (46.8, 59.3) | 36.3 (29.2, 43.4) | 58.3 (53.1, 63.5) | 26.6 (21.4, 31.9) |  |
| Highest Level of Education, % (95% CI) | |  |  |  |  |  | <.001 |
|  | High School or Less | 21.5 (16.8, 26.2) | 14.9 (10.5, 19.4) | 16.2 (10.9, 21.4) | 27.0 (22.1, 31.9) | 15.4 (11.3, 19.5) |  |
|  | Some College or Associates Degree | 34.6 (29.3, 39.9) | 36.3 (30.3, 42.3) | 32.0 (25.3, 38.7) | 41.8 (36.4, 47.2) | 25.8 (20.6, 31.0) |  |
|  | Bachelor’s Degree or Trade School | 30.2 (25.3, 35.1) | 37.0 (30.8, 43.1) | 28.2 (22.2, 34.2) | 22.5 (17.8, 27.1) | 22.7 (18.2, 27.1) |  |
|  | Graduate Degree | 13.7 (10.0, 17.4) | 11.8 (7.7, 15.9) | 23.6 (18.3, 28.9) | 8.7 (5.7, 11.6) | 36.1 (31.0, 41.2) |  |
| Current Healthcare Professional, % (95% CI) | | 9.1 (5.9, 12.2) | 6.8 (3.8, 9.9) | 12.1 (8.3, 15.9) | 4.9 (2.9, 6.9) | 25.1 (20.5, 29.8) | <.001 |
| Current Student, % (95% CI) | | 17.0 (12.3, 21.7) | 20.0 (14.4, 25.7) | 19.4 (13.5, 25.3) | 16.1 (11.4, 20.7) | 21.3 (16.7, 25.8) | >.99 |
| Current or Former Armed Forces, % (95% CI) | | 4.6 (2.7, 6.6) | 4.3 (2.0, 6.7) | 11.5 (7.2, 15.8) | 5.5 (3.4, 7.6) | 16.2 (12.0, 20.4) | <.001 |
| **Health Factors in the Last 12 Months** | | | | | | | |
| Tobacco Use, % (95% CI) | | 30.2 (26.0, 34.5) | 29.8 (24.9, 34.7) | 52.7 (45.7, 59.6) | 58.7 (52.9, 64.5) | 69.6 (63.7, 75.6) | <.001 |
| General Health Self- Assessment, % (95% CI) | | |  |  |  | | <.001 |
|  | Poor or Fair | 12.6 (9.4, 15.9) | 9.4 (6.1, 12.6) | 10.2 (6.3, 14.2) | 17.6 (13.7, 21.6) | 4.7 (2.4, 7.1) |  |
|  | Good | 40.5 (35.2, 45.9) | 36.3 (30.4, 42.3) | 27.7 (21.5, 33.9) | 36.1 (30.9, 41.2) | 22.1 (17.6, 26.6) |  |
|  | Very Good or Excellent | 46.8 (41.3, 52.4) | 54.3 (48.1, 60.5) | 62.1 (55.5, 68.7) | 46.3 (40.8, 51.8) | 73.2 (68.3, 78.0) |  |
| Received Treatment or Counseling, % (95% CI) | |  |  |  |  |  |  |
|  | Alcohol Use | 2.4 (1.3, 3.6) | 2.8 (0.8, 4.9) | 12.2 (7.9, 16.5) | 5.7 (3.4, 7.9) | 22.5 (18.1, 26.9) | <.001 |
|  | Prescription or Illicit Drug Use | 6.5 (4.1, 9.0) | 7.2 (4.4, 10.1) | 12.2 (8.1, 16.4) | 15.7 (12.3, 19.2) | 20.9 (16.7, 25.0) | <.001 |
| Lifetime Mental Health Diagnoses, % (95% CI) | | |  |  |  |  |  |
|  | Attention Deficit/Hyperactivity Disorder | 43.1 (37.7, 48.5) | 20.4 (15.4, 25.5) | 26.0 (19.8, 32.3) | 24.2 (19.6, 28.8) | 29.4 (24.4, 34.3) | <.001 |
|  | Any Anxiety Disorder | 41.6 (36.2, 46.9) | 43.5 (37.2, 49.7) | 37.6 (30.9, 44.3) | 44.9 (39.5, 50.3) | 39.8 (34.5, 45.2) | >.99 |
|  | Autism Spectrum Disorder | 2.4 (0.8, 4.0) | 1.2 (0.0, 2.5) | 4.9 (1.6, 8.2) | 0.9 (0.1, 1.7) | 14.2 (10.1, 18.2) | <.001 |
|  | Bipolar Disorder | 13.1 (9.8, 16.5) | 12.3 (8.4, 16.3) | 9.6 (6.1, 13.0) | 18.4 (14.6, 22.2) | 20.1 (15.7, 24.4) | .02 |
|  | Borderline Personality Disorder | 3.7 (1.7, 5.7) | 4.8 (2.2, 7.3) | 2.9 (1.2, 4.6) | 5.5 (3.3, 7.7) | 6.0 (3.9, 8.2) | >.99 |
|  | Eating Disorder | 5.5 (3.0, 8.1) | 2.9 (1.0, 4.8) | 6.5 (3.2, 9.8) | 3.7 (1.6, 5.7) | 9.1 (5.9, 12.2) | .10 |
|  | Major Depressive Disorder | 20.2 (16.1, 24.4) | 17.8 (13.1, 22.5) | 12.1 (7.6, 16.6) | 23.1 (18.7, 27.5) | 11.4 (7.8, 15.0) | .007 |
|  | Post-Traumatic Stress Disorder | 9.5 (6.6, 12.4) | 12.6 (8.7, 16.6) | 9.4 (5.3, 13.4) | 18.2 (14.1, 22.3) | 9.0 (5.9, 12.2) | 0.01 |
|  | Schizophrenia | 2.4 (0.9, 4.0) | 1.6 (0.2, 3.0) | 2.7 (0.3, 5.1) | 2.7 (1.0, 4.4) | 3.7 (1.8, 5.7) | >.99 |
| Drug Abuse Screening Test, 10-item Score, Mean 95% CI | | 1.7 (1.5, 1.9) | 2.2 (1.9, 2.4) | 2.6 (2.2, 2.9) | 4.2 (3.9, 4.5) | 4.2 (3.9, 4.5) | <.001 |
| Non-Pharmaceutical Stimulant Use, % (95% CI) | |  |  |  |  |  |  |
|  | Cocaine | 3.6 (2.0, 5.2) | 21.7 (16.5, 26.9) | 11.1 (6.4, 15.7) | 33.8 (28.7, 39.0) | 24.9 (20.4, 29.4) | <.001 |
|  | Methamphetamine | 2.3 (1.0, 3.5) | 8.3 (5.3, 11.3) | 12.1 (7.8, 16.4) | 31.7 (27.0, 36.4) | 28.2 (23.4, 33.1) | <.001 |
|  | MDMA^b^ | 4.4 (2.3, 6.5) | 10.7 (6.6, 14.8) | 8.0 (4.4, 11.7) | 20.9 (16.4, 25.5) | 22.0 (17.6, 26.5) | <.001 |
|  | Illicit Amphetamine | 1.4 (0.2, 2.5) | 2.0 (0.6, 3.5) | 7.7 (4.2, 11.1) | 12.1 (8.8, 15.4) | 26.6 (21.9, 31.3) | <.001 |
| Opioid Use, % (95% CI) | |  |  |  |  |  |  |
|  | Non-medical Use Low Potency Opioids | 21.7 (17.4, 26.0) | 17.6 (13.1, 22.1) | 29.9 (23.7, 36.1) | 29.5 (24.7, 34.3) | 37.8 (32.6, 43.0) | <.001 |
|  | Non-medical Use Medium Potency Opioids | 27.4 (22.6, 32.2) | 23.0 (18.0, 27.9) | 30.8 (24.7, 36.8) | 43.3 (38.0, 48.7) | 36.9 (31.5, 42.3) | <.001 |
|  | Non-medical Use High Potency Opioids | 5.6 (3.1, 8.0) | 3.3 (1.4, 5.2) | 19.6 (14.3, 24.8) | 15.9 (12.4, 19.5) | 26.9 (22.3, 31.6) | <.001 |
|  | Non-medical Use Maintenance Opioids | 6.6 (4.1, 9.1) | 4.0 (1.9, 6.1) | 19.8 (14.5, 25.1) | 18.7 (14.9, 22.6) | 18.6 (14.8, 22.5) | <.001 |
|  | Non-pharmaceutical Opioid Use | 1.7 (0.7, 2.7) | 2.8 (1.1, 4.5) | 9.5 (6.0, 13.1) | 15.5 (12.0, 19.0) | 29.2 (24.5, 34.0) | <.001 |

^a^ Respondents may select more than one race

^b^3,4 methylenedioxymethamphetamine

**Table S5: Latent Class Modeling Results – Non-Medical Use of Prescription Stimulants**

| **Latent Classes** | **Increase in Mean DAST-10 Score** | | | **Drug Use Treatment/Counseling in Last 12 Months** | | |
| --- | --- | --- | --- | --- | --- | --- |
|  | **Crude 95% CI** | **Adjusted^a^**  **95% CI** | **Adjusted^a^ p-value** | **Crude OR (95% CI)** | **Adjusted^b^**  **OR (95% CI)** | **Adjusted^b^ p-value** |
| Non-Medical Use of Prescription Stimulants | | | | | | |
| Amphetamine Self-Medication | REF | REF | - | REF | REF | - |
| Network Sourced for Alertness | 0.49 (0.15, 0.83) | 0.48 (0.15, 0.82) | 0.005 | 1.12 (0.62, 2.00) | 1.12 (0.62, 2.00) | 0.714 |
| Non-Amphetamine Performance Use | 0.88 (0.48, 1.27) | 0.68 (0.27, 1.08) | 0.001 | 2.00 (1.14, 3.48) | 1.70 (0.95, 3.04) | 0.074 |
| Recreational Use | 2.48 (2.10, 2.85) | 2.19 (1.81, 2.57) | <0.001 | 2.67 (1.66, 4.32) | 2.23 (1.35, 3.67) | 0.002 |
| Non-Discriminatory Behaviors | 1.97 (1.63, 2.31) | 1.63 (1.26, 1.99) | <0.001 | 3.77 (2.35, 6.06) | 2.87 (1.71, 4.83) | <0.001 |

**Table S6: Latent Class Modeling Results – Non-Medical Use of Prescription Opioids**

| **Model Parameters** | | **Moderate Pain with Low Mental Health Burden** | **High Pain with Higher Mental Health Burden** | **Risky Behaviors Diverse Motivations** | **Non-Discriminatory Behaviors** |
| --- | --- | --- | --- | --- | --- |
| Prevalence | | 0.22 (0.01) | 0.38 (0.01) | 0.21 (0.02) | 0.19 (0.02) |
| Drug Use | |  |  |  |  |
|  | Low-Potency Opioid NMU | **1.00 (0.00)** | 0.33 (0.01) | 0.37 (0.03) | **0.57 (0.02)** |
|  | Medium-Potency Opioid NMU | 0.00 (0.00) | **1.00 (0.00)** | 0.48 (0.03) | **0.66 (0.02)** |
|  | High-Potency Opioid NMU | 0.00 (0.00) | 0.03 (0.01) | 0.23 (0.02) | 0.36 (0.02) |
|  | Maintenance Opioid NMU | 0.00 (0.00) | 0.00 (0.00) | 0.28 (0.02) | 0.30 (0.02) |
|  | Any Recreational Opioid | 0.00 (0.00) | 0.01 (0.00) | 0.11 (0.02) | 0.29 (0.02) |
| Reason for Prescription NMU | |  |  |  |  |
|  | To Reduce Pain | **0.76 (0.02)** | **0.85 (0.01)** | **0.62 (0.02)** | **0.83 (0.02)** |
|  | Medical Condition or Symptom Other Than Pain | 0.18 (0.01) | 0.18 (0.01) | 0.30 (0.02) | **0.61 (0.03)** |
|  | Enjoyment/To Get High | 0.08 (0.01) | 0.18 (0.01) | 0.25 (0.05) | **0.74 (0.02)** |
|  | To Relax/Reduce Stress or Sleep | 0.31 (0.02) | 0.43 (0.01) | 0.39 (0.03) | **0.81 (0.02)** |
|  | To Prevent or Treat Withdrawal Symptoms/ Come Down from a High | 0.03 (0.01) | 0.05 (0.01) | 0.25 (0.03) | **0.83 (0.03)** |
| Route of Administration for Prescription NMU | |  |  |  |  |
|  | Swallowed | **0.98 (0.01)** | **1.00 (0.01)** | **0.53 (0.04)** | **0.90 (0.02)** |
|  | Other Oral | 0.04 (0.01) | 0.07 (0.01) | **0.52 (0.02)** | **0.83 (0.03)** |
|  | Nonoral - Inhaled/Injected | 0.00 (0.00) | 0.03 (0.01) | 0.32 (0.03) | **0.81 (0.02)** |
| Source of Drug for Prescription NMU | |  |  |  |  |
|  | Valid Prescription | **0.50 (0.02)** | **0.56 (0.01)** | 0.46 (0.03) | **0.69 (0.03)** |
|  | Friend or Family Member | 0.40 (0.02) | **0.50 (0.01)** | 0.37 (0.03) | **0.77 (0.02)** |
|  | Dealer | 0.04 (0.01) | 0.06 (0.01) | 0.17 (0.04) | **0.61 (0.02)** |
|  | Other Diversion excluding Online | 0.10 (0.01) | 0.07 (0.01) | 0.29 (0.02) | **0.66 (0.03)** |

Data shown as probability (standard error); item-response probabilities >0.5 bolded to facilitate interpretation

**Table S7: Characteristics among Non-Medical Use of Prescription Opioids Latent Classes**

| **Characteristic** | | | **Moderate Pain with Low Mental Health Burden** | | **High Pain with Higher Mental Health Burden** | | **Risky Behaviors**  **Diverse Motivations** | **Non-Discriminatory Behaviors** | | **Holm P-value** |
| --- | --- | --- | --- | --- | --- | --- | --- | --- | --- | --- |
| Unweighted N | | 1,218 | | 2,317 | | 1,215 | | | 1,377 | - |
| **Demographics** | | | | | | | | | | |
| Age (years), Mean (95% CI) | | | 41.4 (40.4, 42.5) | | 41.4 (40.6, 42.1) | | 35.4 (34.5, 36.3) | 33.1 (32.4, 33.7) | | <.001 |
| Sex: Male, % (95% CI) | | | 52.0 (48.6, 55.3) | | 50.1 (47.7, 52.5) | | 65.6 (62.4, 68.9) | 71.3 (68.5, 74.2) | | <.001 |
| Race^a^, % (95% CI) | | |  | |  | |  |  | |  |
|  | American Indian orAlaska Native | | 2.4 (1.4, 3.4) | | 2.0 (1.3, 2.6) | | 3.1 (1.8, 4.4) | 3.0 (1.8, 4.2) | | >.99 |
|  | Asian | | 7.2 (5.2, 9.1) | | 3.5 (2.6, 4.5) | | 6.3 (4.6, 8.0) | 6.7 (4.8, 8.5) | | .005 |
|  | Black or African American | | 15.5 (12.9, 18.0) | | 15.7 (13.8, 17.6) | | 16.8 (13.9, 19.6) | 17.5 (14.8, 20.2) | | >.99 |
|  | Native Hawaiian or Pacific Islander | | Suppressed | | 0.7 (0.3, 1.2) | | 1.1 (0.4, 1.8) | 1.0 (0.4, 1.6) | | >.99 |
|  | White | | 72.4 (69.2, 75.5) | | 76.8 (74.6, 78.9) | | 68.9 (65.5, 72.3) | 71.8 (68.7, 75.0) | | .005 |
|  | Other Race | | 6.0 (4.3, 7.8) | | 4.2 (3.1, 5.2) | | 6.0 (4.2, 7.9) | 3.4 (2.1, 4.7) | | .22 |
| Ethnicity, % (95% CI) | | |  | |  | |  |  | |  |
|  | Hispanic/Latinx | | 17.3 (14.6, 20.0) | | 11.6 (10.0, 13.3) | | 16.9 (14.3, 19.6) | 18.0 (15.4, 20.5) | | <.001 |
| Annual Household Income, % (95% CI) | | |  | |  | |  |  | | .06 |
|  | <$50,000 | | 49.9 (46.6, 53.3) | | 49.1 (46.7, 51.6) | | 46.7 (43.2, 50.2) | 42.8 (39.5, 46.1) | |  |
|  | $50,000 - $74,999 | | 35.0 (31.8, 38.1) | | 36.5 (34.1, 38.8) | | 38.8 (35.4, 42.2) | 38.2 (35.0, 41.3) | |  |
|  | >$75,000 | | 15.1 (12.8, 17.4) | | 14.4 (12.7, 16.0) | | 14.5 (12.3, 16.7) | 19.0 (16.6, 21.5) | |  |
| Marital Status, % (95% CI) | | |  | |  | |  |  | | >.99 |
|  | Married | | 48.7 (45.4, 52.1) | | 47.2 (44.8, 49.6) | | 46.8 (43.3, 50.2) | 47.9 (44.7, 51.2) | |  |
|  | Never Married | | 16.2 (14.0, 18.5) | | 18.2 (16.5, 19.9) | | 14.9 (12.6, 17.2) | 17.0 (14.6, 19.4) | |  |
|  | Widowed or Divorced or Separated | | 35.1 (31.7, 38.4) | | 34.6 (32.2, 37.0) | | 38.3 (34.8, 41.8) | 35.1 (31.9, 38.4) | |  |
| Highest Level of Education, % (95% CI) | | |  | |  | |  |  | | <.001 |
|  | High School or Less | | 22.3 (19.5, 25.2) | | 24.4 (22.3, 26.6) | | 23.3 (20.3, 26.3) | 23.6 (20.7, 26.5) | |  |
|  | Some College or Associates Degree | | 33.7 (30.5, 36.9) | | 38.5 (36.1, 40.8) | | 34.5 (31.1, 37.8) | 30.7 (27.6, 33.8) | |  |
|  | Bachelor’s Degree or Trade School | | 32.2 (29.1, 35.3) | | 27.5 (25.3, 29.6) | | 26.6 (23.5, 29.7) | 25.0 (22.2, 27.8) | |  |
|  | Graduate Degree | | 11.7 (9.7, 13.8) | | 9.6 (8.2, 11.0) | | 15.6 (13.3, 17.9) | 20.7 (18.3, 23.1) | |  |
| Current Healthcare Professional, % (95% CI) | | | 5.4 (3.9, 7.0) | | 5.4 (4.2, 6.5) | | 10.0 (8.0, 12.1) | 15.0 (12.9, 17.2) | | <.001 |
| Current Student, % (95% CI) | | | 9.1 (6.8, 11.4) | | 9.3 (7.6, 11.0) | | 14.5 (11.8, 17.2) | 17.0 (14.4, 19.7) | | <.001 |
| Current or Former Armed Forces, % (95% CI) | | | 6.4 (5.0, 7.9) | | 7.3 (6.1, 8.4) | | 8.8 (7.0, 10.5) | 11.4 (9.4, 13.4) | | 0.002 |
| **Health Factors in the Last 12 Months** | | | | | | | | | | |
| Tobacco Use, % (95% CI) | | | 23.7 (21.4, 26.1) | | 31.6 (29.7, 33.6) | | 47.8 (44.3, 51.2) | 64.3 (60.8, 67.9) | | <.001 |
| General Health Self-Assessment, % (95% CI) | | |  | |  | |  |  | | <.001 |
|  | Poor or Fair | | 16.9 (14.6, 19.2) | | 18.3 (16.6, 20.0) | | 14.5 (12.2, 16.9) | 13.0 (10.9, 15.2) | |  |
|  | Good | | 39.3 (36.0, 42.6) | | 41.3 (38.9, 43.7) | | 35.2 (31.9, 38.5) | 28.3 (25.4, 31.2) | |  |
|  | Very Good or Excellent | | 43.8 (40.5, 47.2) | | 40.4 (38.0, 42.8) | | 50.3 (46.8, 53.8) | 58.7 (55.5, 61.8) | |  |
| Received Treatment or Counseling, % (95% CI) | | |  | |  | |  |  | |  |
|  | Alcohol Use | | 2.2 (1.4, 2.9) | | 2.7 (2.0, 3.5) | | 7.1 (5.5, 8.6) | 13.0 (11.1, 14.9) | | <.001 |
|  | Prescription or Illicit Drug Use | | 2.6 (1.6, 3.7) | | 3.2 (2.4, 4.0) | | 12.7 (10.5, 14.9) | 25.7 (23.0, 28.4) | | <.001 |
| Pain Experience, % (95% CI) | | |  | |  | |  |  | |  |
|  | Acute or Chronic Pain | | 50.9 (47.5, 54.3) | | 60.0 (57.5, 62.4) | | 45.1 (41.6, 48.5) | 55.2 (51.9, 58.5) | | <.001 |
|  | Acute or Chronic & Saw Physician | | 37.7 (34.5, 40.8) | | 44.2 (41.8, 46.5) | | 31.8 (28.7, 34.8) | 31.6 (28.7, 34.4) | | <.001 |
|  | Acute or Chronic & Received Pain Reliever Prescription | | 26.8 (24.0, 29.6) | | 34.1 (31.9, 36.3) | | 25.5 (22.7, 28.3) | 25.6 (22.9, 28.3) | | <.001 |
| Mental Health Diagnoses, % (95% CI) | | |  | |  | |  |  | |  |
|  | Attention Deficit /Hyperactivity Disorder | | 7.0 (5.2, 8.8) | | 9.1 (7.7, 10.5) | | 13.9 (11.6, 16.2) | 24.8 (22.0, 27.6) | | <.001 |
|  | Any Anxiety Disorder | | 29.7 (26.7, 32.7) | | 37.7 (35.3, 40.0) | | 29.2 (26.2, 32.2) | 40.6 (37.4, 43.7) | | <.001 |
|  | Autism Spectrum Disorder | | 1.4 (0.5, 2.4) | | 0.8 (0.3, 1.3) | | 2.5 (1.3, 3.8) | 7.6 (5.8, 9.3) | | <.001 |
|  | Bipolar Disorder | | 7.3 (5.6, 8.9) | | 9.6 (8.3, 10.9) | | 12.3 (10.1, 14.4) | 22.9 (20.2, 25.5) | | <.001 |
|  | Borderline Personality Disorder | | 1.5 (0.8, 2.3) | | 2.3 (1.6, 3.0) | | 4.3 (3.0, 5.7) | 8.3 (6.6, 10.0) | | <.001 |
|  | Eating Disorder | | 2.7 (1.6, 3.9) | | 2.9 (2.1, 3.6) | | 4.5 (3.1, 5.8) | 9.6 (7.5, 11.8) | | <.001 |
|  | Major Depressive Disorder | | 14.2 (11.9, 16.5) | | 15.0 (13.3, 16.6) | | 11.5 (9.5, 13.5) | 14.1 (12.0, 16.1) | | .50 |
|  | Post-Traumatic Stress Disorder | | 6.7 (5.2, 8.2) | | 9.5 (8.2, 10.8) | | 9.8 (7.9, 11.7) | 15.1 (12.8, 17.3) | | <.001 |
|  | Schizophrenia | | 1.1 (0.3, 1.8) | | 1.4 (0.9, 2.0) | | 1.8 (0.9, 2.7) | 4.1 (2.9, 5.4) | | <.001 |
| Drug Abuse Screening Test, 10-item Score, Mean (95% CI) | | | 1.1 (1.0, 1.2) | | 1.6 (1.5, 1.7) | | 2.4 (2.3, 2.6) | 4.0 (3.8, 4.2) | | <.001 |
| Non-Pharmaceutical Opioid Use, % (95% CI) | | |  | |  | |  |  | |  |
|  | Heroin | | 0.1 (0.0, 0.2) | | 0.6 (0.3, 0.9) | | 8.1 (6.3, 9.8) | 21.0 (18.5, 23.5) | | <.001 |
|  | Fentanyl | | Suppressed | | Suppressed | | 5.4 (4.0, 6.8) | 18.4 (16.0, 20.8) | | <.001 |
| Stimulant Use, % (95% CI) | | |  | |  | |  |  | |  |
|  | Non-medical Use Amphetamine | | 5.1 (3.7, 6.4) | | 10.2 (8.7, 11.7) | | 16.0 (13.5, 18.4) | 23.9 (21.2, 26.6) | | <.001 |
|  | Non-Medical Use Methylphenidate | | 0.5 (0.2, 0.9) | | 1.5 (1.0, 2.1) | | 6.3 (4.6, 8.0) | 10.3 (8.4, 12.1) | | <.001 |
|  | Non-medical Use Modafinil | | 0.4 (0.0, 0.8) | | 0.8 (0.4, 1.3) | | 4.4 (3.2, 5.7) | 9.9 (8.2, 11.6) | | <.001 |
|  | Non-pharmaceutical Stimulant Use | | 7.0 (5.3, 8.7) | | 11.0 (9.5, 12.4) | | 22.8 (20.1, 25.6) | 47.5 (44.3, 50.8) | | <.001 |

^a^ Respondents may select more than one race

**Table S8: Latent Class Modeling Results – Non-Medical Use of Prescription Opioids**

| **Latent Classes** | **Increase in Mean DAST-10 Score** | | | **Drug Use Treatment/Counseling in Last 12 Months** | | |
| --- | --- | --- | --- | --- | --- | --- |
|  | **Crude 95% CI** | **Adjusted^a^**  **95% CI** | **Adjusted^a^ p-value** | **Crude OR (95% CI)** | **Adjusted^b^**  **OR (95% CI)** | **Adjusted^b^ p-value** |
| Non-Medical Use of Prescription Opioids | | | | | | |
| Moderate Pain with Low Mental Health Burden | REF | REF | - | REF | REF | - |
| High Pain with Higher Mental Health Burden | 0.50 (0.37, 0.63) | 0.35 (0.21, 0.48) | <0.001 | 1.23 (0.76, 1.99) | 1.07 (0.66, 1.74) | <0.001 |
| Risky Behaviors Diverse Motivations | 1.33 (1.15, 1.52) | 1.08 (0.90, 1.26) | <0.001 | 5.40 (3.43, 8.49) | 4.91 (3.09, 7.80) | <0.001 |
| Non-Discriminatory Behaviors | 2.89 (2.68, 3.09) | 2.36 (2.15, 2.58) | <0.001 | 12.84 (8.34, 19.78) | 9.74 (6.22, 15.26) | <0.001 |
