# Supplementary material for: Differing Behaviors Around Adult Nonmedical Use of Prescription Stimulants and Opioids: Latent Class Analysis
Source: J Med Internet Res. 2023 Sep 20;25:e46742. doi: 10.2196/46742 (PMC10551786; doi:10.2196/46742)
Supplement: Multimedia Appendix 2 [file jmir_v25i1e46742_app2.docx]

Multimedia Appendix 2: Consent Language

This year, we are surveying about 60,000 people across the country. You are asked to take part in this research study. You will represent other people in the United States who are similar to you. This study asks about your use of medications, tobacco, alcohol, drugs, and other health issues. Your answers about these topics are important. Policymakers and researchers can use information from this study to understand drug addiction and treatment needs in the United States. This survey should take about 10 to 15 minutes to complete.

Taking this survey is voluntary. You do not have to take the survey. If you start to take the survey and change your mind, you may stop. We will only use information you choose to enter into the survey before stopping. There will not be any penalty for not taking the survey or for stopping the survey. You will be paid according to your panel’s policy. We ask that you try to answer all questions. Some questions and sections are designed so that you do not have to answer.

Your answers will be completely confidential and are anonymous to the researchers. The researchers will not receive any information that can identify you. The survey panel that gives this survey will link a code to you so that the researchers can note if you have taken more than one survey. However, the researchers will never be able to identify you with this code.

This research is covered by a Certificate of Confidentiality from the National Institutes of Health. The researchers with this Certificate may not disclose or use information or documents that may identify you in any federal, state, or local civil, criminal, administrative, legislative, or other action, suit, or proceeding, or be used as evidence, for example, if there is a court subpoena, unless you have consented for this use. Information protected by this Certificate cannot be disclosed to anyone else who is not connected with the research except if it is required by federal, state, or local laws, or used for other scientific research, as allowed by federal regulations protecting research subjects. You should understand that a Certificate of Confidentiality does not prevent you from voluntarily releasing information or documents about yourself or your involvement in this research. These protections apply only to your research records.

I was provided with information about the survey and I was told that information I provide will be kept private. I choose to take the survey. Answer: Yes/No.
